# Foodborne cereulide causes beta cell dysfunction and apoptosis

**DOI:** 10.1186/2049-3258-72-S1-O8

**Published:** 2014-06-06

**Authors:** Roman Vangoitsenhoven, Dieter Rondas, Inne Crèvecoeur, Wannes D'Hertog, Matilde Masini, Mirjana Andjelkovic, Joris Van Loco, Christophe Matthys, Chantal Mathieu, Lut Overbergh, Bart Van der Schueren

**Affiliations:** 1 Clinical and Experimental Medicine and Endocrinology, KU Leuven, Leuven, Belgium; 2 Department of Translational Research and of New Surgical and Medical Technologies, University of Pisa, Italy; 3 Food, Medicines, and Consumer Safety, Scientific Institute of Public Health, Brussels, Belgium

## Background

Environmental factors play a major role in the rising prevalence of type 1 and type 2 diabetes mellitus. Cereulide is a lipophilic peptide that is often found at low concentrations in starchy food. It is a culprit to consider in this era of prepackaged meals.

## Materials and methods

Mouse and rat insulin producing beta cell lines, MIN6 and INS-1E respectively, as well as whole mouse islets, isolated from 2 week old C57Bl/6J mice, were exposed to cereulide concentrations ranging from 0.05ng/ml to 5ng/ml for 24 and 72h. Cell death was evaluated by a Hoechst/Propidium Iodide assay, and compared to cell death in human hepatocellular HepG2 and monkey fibroblast-like COS-1 cells. Subsequently, MIN6 cells were exposed to low concentrations of cereulide (0.15 - 0.5 ng/ml) for 24h and glucose-stimulated insulin secretion was evaluated as well as mechanisms of toxicity by mRNA profiling, electron microscopy and caspase activation and cytochrome c release assay.

## Results

Cereulide exposure caused cell death in MIN6, INS-1E and pancreatic islets, but not in HepG2 or COS-1E cells (Table 1). Caspase 3/7 activation confirmed the apoptotic cell death process. Glucose-stimulated insulin secretion decreased from 10.48 ± 3.33 fold to 2.01 ± 0.51 (P < 0.05) in MIN6 cells after 24h exposure with 0.25 ng/ml cereulide. Exposure to 0.25ng/ml cereulide induced markers of mitochondrial stress, including PUMA (p53 upregulated modulator of apoptosis; 271 ± 77 % of control; P < 0.05) but also markers of ER stress, such as CHOP (CCAAT/-enhancer-binding protein homologous protein; 641 ± 190 % of control; P < 0.01). EM revealed swelling and loss of mitochondria, and cytoplasmic cytochrome c release confirmed mitochondrial cell death signalling (360 ± 83 % of control after exposure to 0.5 ng/ml for 24h (P < 0.05).

**Table 1:** Apoptosis induced after 24h exposure to cereulide (mean percentage ± SEM).

	MIN6 (n=5)	INS-1E (n=4)	HepG2 (n=3)	COS (n=3)	Islets (n=3)
Medium	7.3 ± 1.3	2.5 ± 0.3	5.8 ± 0.6	1.2 ± 0.6	3.1 ± 1.2

0.05 ng/ml cereulide	5.9 ± 1.0	3.2 ± 0.5	6.6 ± 2.1	1.6 ± 0.4	3.9 ± 1.5

0.25 ng/ml cereulide	31.6 ± 5.8 *	58.1 ± 11.4 *	6.9 ± 1.5	2.9 ± 0.7	8.6 ± 2.4

0.5 ng/ml cereulide	43.6 ± 6.1 *	100.0 ± 0.0 *	11.9 ± 2.5	2.6 ± 0.6	49.2 ± 9.0

5 ng/ml cereulide	100.0 ± 0.0 *	100.0 ± 0.0 *	7.7 ± 2.3	4.3 ± 0.9	96.4 ± 3.5*

* p ≤ 0.05 vs control

## Conclusion

Cereulide, a toxin frequently found in prepackaged or prepared starchy meals, increases levels of mitochondrial and ER stress markers in beta cells of rats and mice, even at low doses. In a dose dependent way, it also leads to impaired beta cell function and apoptosis. Cereulide might thus be involved in the current diabetes.

